# Targeting SARS-CoV-2 main protease (3CL^pro^) with Paeonia-derived phytochemicals

**DOI:** 10.1007/s40203-026-00630-7

**Published:** 2026-04-17

**Authors:** Cemal Sandalli, Safiye Merve Bostancioglu, Aytul Sandalli, Emine Akyuz Turumtay, Dana Almohazey, Moneerah Alsaeed, Galyah Alhamid, Ali A. Rabaan, Halbay Turumtay, Ei-ichi Ami, Christian L. Lorson, Mark Hannink, Kamal Singh, Huseyin Tombuloglu

**Affiliations:** 1 Christopher Bond Life Sciences Center, University of Missouri, Columbia, MO 65211 USA; 2 Department of Biological Sciences, Middle East Technical University, 06800 Ankara, Türkiye; 3 Department of Biology, Karadeniz Technical University, 61080 Trabzon, Türkiye; 4 Department of Chemistry, Recep Tayyip Erdogan University, 53100 Rize, Türkiye; 5 Technology Division, Joint BioEnergy Institute, Emeryville, CA 94608 USA; 6 Department of Stem Cell Research, Institute for Research and Medical Consultations, Imam Abdulrahman Bin Faisal University, P.O. Box 1982, 31441 Dammam, Saudi Arabia; 7 Department of Genetics Research, Institute for Research and Medical Consultations (IRMC), Imam Abdulrahman Bin Faisal University, P.O. Box 1982, 31441 Dammam, Saudi Arabia; 8 Molecular Diagnostic Laboratory, Johns Hopkins Aramco Healthcare, Dhahran, 31311 Saudi Arabia; 9 College of Medicine, Alfaisal University, Riyadh, 11533 Saudi Arabia; 10 Department of Public Health and Nutrition, The University of Haripur, Haripur, 22610 Pakistan; 11 Department of Energy System Engineering, Karadeniz Technical University, 61830 Trabzon, Türkiye; 12 Deparment of Molecular and Cell Biology, University of California, Berkeley, Berkeley, CA 94720 USA; 13 Department of Pathobiology and Integrative Biomedical Sciences, University of Missouri, Columbia, MO 65211 USA; 14 Division of Clinical Microbiology, Department of Laboratory Medicine, Karolinska Institute, 14186 Stockholm, Sweden

**Keywords:** SARS-CoV-2; SARS-CoV-2, 3CL^pro^ protease, Paeonia, Phytochemicals, Paeoniflorigenone

## Abstract

Coronavirus Disease 19 (COVID-19), caused by Severe Acute Respiratory Syndrome Coronavirus 2 (SARS-CoV-2), stimulated intensive drug-development efforts throughout the world. As COVID-19 has become endemic in many regions of the world, phytochemicals used in ethnomedicine may have utility in the prevention or treatment of COVID-19. Here, we employed machine learning/deep learning drug discovery tools to evaluate compounds previously identified in roots of plants of the Paeonia genus for their drug-like properties and potential to inhibit 3CL^pro^, the main protease of SARS-CoV2. Our results identify Paeonia-derived compounds that have favorable drug-like properties and are predicted to have a high binding affinity for 3CL^pro^. Molecular dynamics simulations supported the binding affinity of Paeonia-derived compounds for 3CL^pro^. We validated these in silico computational results by experimentally determining the 50% inhibitory concentration (IC_50_) of two Paeonia-derived compounds, paeoniflorigenone and 3-*O*-methylquercetin, using purified 3CL^pro^ with baicalein as a control. The IC_50_ values for paeoniflorigenone, 3-*O*-methylquercetin and baicalein were 9.33 µM, 43.94 and 43.85 µM, respectively. Root extracts from five Paeonia species were found to have minimal cytotoxicity against three different human cell types. Our results represent a proof-of-concept study demonstrating that in silico techniques, including machine and deep learning methods, can be used to identify phytochemicals as starting points for the discovery of antiviral compounds against SARS-CoV-2.

**Supplementary Information:**

The online version contains supplementary material available at10.1007/s40203-026-00630-7.

## Introduction

More than seven million people across the world have died from Coronavirus Disease 19 (COVID-19) since December 2019 (Sachs et al. 2022; WHO, (https://data.who.int/dashboards/covid19/cases). COVID-19 is caused by Severe Acute Respiratory Syndrome Coronavirus 2 (SARS-CoV-2), which belongs to the *Betacoronavirus* of the Coronaviridae family (Amoutzias et al. 2022; Hu et al. 2021). SARS-CoV-2 shares ~ 79% genomic sequence identity with SARS-CoV (Hu et al. 2021), which emerged in 2002–2003, and ~ 50% genomic sequence identity with Middle East Respiratory Coronavirus (MERS-CoV), which emerged in 2012 (Zaki et al. 2012). The Coronaviruses are single-stranded positive-sense RNA genome viruses. The SARS-CoV-2 genome has six open reading frames (ORFs) that encode 6 polypeptides, including two large non-structural polypeptides pp1a and pp1ab, spike (S), envelope (E), membrane (M), and nucleocapsid (N). Seven putative ORFs, which encode accessory proteins interspersed between the structural genes, have also been identified in SARS-CoV-2 (Jungreis et al. 2021). The polypeptides pp1a and pp1ab contain two viral proteases: (i) papain-like protease (PL^pro^) and (ii) 3CL^pro^ (also known as the main protease, or M^pro^). These proteases cleave pp1a and pp1ab into 16 non-structural proteins (nsps). These nsps include single-stranded RNA binding protein (nsp9), cofactor for nsp14 (nsp10), viral RNA-dependent RNA polymerase (nsp12), RNA helicase (nsp13), exoribonuclease (nsp14), endoribonuclease (nsp15), and 2´-O-ribose methyltransferase (nsp16) forming a replication-transcription complex that plays an essential role in the viral replication cycle (Yan et al. 2022). Rapid progress in understanding how these viral proteins contribute to the lifecycle of SARS-CoV-2 has enabled the development of vaccines and small-molecule drugs that are both safe and effective against COVID-19 (Hogan and Pardi 2022; Li et al. 2023). However, the worldwide endemic presence of SARS-CoV-2 and the emergence of mutant variants that have acquired resistance to one or more current therapies necessitate the ongoing development of new therapeutics.

The SARS-CoV-2 main protease 3CL^pro^ is a cysteine protease that functions as a homodimer. Each monomer of the homodimer contains three structurally distinct domains: domain 1, domain II, and domain III. Domains I and II assume antiparallel β-barrel structures, and together they form the catalytic active site cleft. Domain III is a helical domain that is presumably involved in regulating dimerization and enzymatic activity. Proteolytic activity is conducted by the H41-C145 catalytic dyad, where H41 acts as a general base activating the thiol group of C145 for nucleophilic attack on the peptide bond, and creating a negatively charged oxygen atom (an oxyanion). The oxyanion hole formed by Gly143, Ser144, and Cys145 stabilizes the transition state during substrate cleavage. The substrate-binding pocket is formed by subsites S1, S2, S4, and S1′, which collectively define ligand specificity and can accommodate a wide range of peptidomimetic and non-peptide inhibitors (Zin et al., 2020). Several residues including H41, C145, G143, S144, H163, H164, E166, and Q189 are involved in inhibitor binding. The high conservation of these residues across coronaviruses and the absence of closely related human homologs make 3CL^pro^ an especially attractive target for structure-based drug discovery.

Ethnomedicine and medicinal herbs have been traditionally used to treat human diseases. Ethnomedicinal herbs have been shown to contain molecules capable of inhibiting SARS-CoV-2 infection of human cells (Jan et al. 2021; Khan et al. 2021; Su et al. 2020). However, the chemical complexity of medicinal herbs is a significant obstacle in ethnobotanical drug discovery. Recent advances in artificial intelligence (AI) and machine-learning (ML) technologies may expedite the identification of plant-based inhibitors of emerging and re-emerging pathogens.

Plants in the genus *Paeonia* are widely distributed in temperate regions around the world and extracts from various *Paeonia* species have been widely used in traditional ethnomedical practices (Li et al. 2021). Both extracts and purified compounds from multiple *Paeonia* species have been shown to have anti-oxidative, anti-inflammatory, anti-microbial, anti-tumor, neuroprotective and cardioprotective activities (He and Dai 2011; Li et al. 2021). For example, paeoniflorigenone, a monoterpene, has been shown to induce apoptosis in tumor-derived cell lines (Huang et al. 2017; Park et al. 2022); and inhibit bacterial and retroviral DNA polymerases (Demir et al. 2019).

Here, we report that Paeonia root extracts have low to moderate toxicity towards human cells and contain compounds able to inhibit the protease activity of 3CL^pro^, the main protease of SARS-CoV2. Paeonia-derived phytochemicals listed in the Natural Product Activity and Species Source (NPASS) (Zhao et al. 2023) databases were screened in silico for their ability to bind 3CL^pro^, using both AutoDock Vina (Eberhardt et al. 2021; Trott and Olson 2010), which uses an empirical scoring function, and GNINA, a deep learning (DL)-based docking tool which uses a convolutional neural network scoring function (McNutt et al. 2021, 2025). The docking scores were combined with in silico assessments of toxicity, synthetic accessibility and lipophilicity to identify compounds with favorable drug-like properties. Two of the Paeonia compounds with favorable drug-like properties were evaluated for their ability to inhibit the protease activity of purified 3CL^pro^. Taken together, our results suggest that the compounds from Paeonia roots may serve as hit compounds for developing safe plant-derived therapeutics against SARS-CoV-2, and likely against future coronavirus outbreaks, as they target a validated and conserved coronavirus target.

## Results

**Fig. 1:**
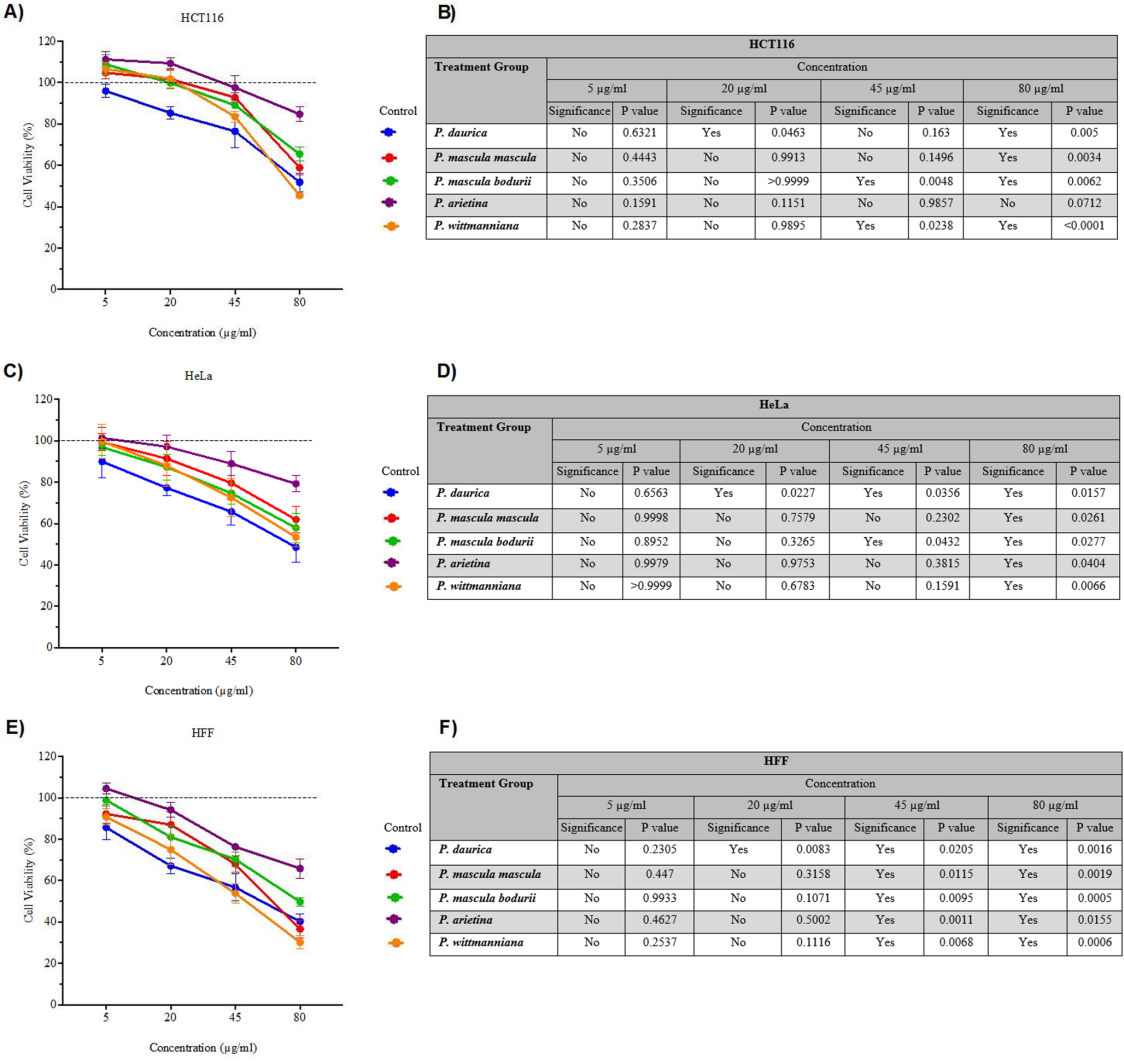
Monolayer cultures of HCT116 (**A**), HeLa (**C**), and HFF (**E**) cells were treated with extracts from indicated Paeonia species, including P. daurica, P. mascula subsp. mascula, P. mascula subsp. bodurii, P. arietina, and P. wittmanniana. The concentration of extracts was 5, 20, 45, and 80 ug/ml. Cell viability was measured at 48 hours with the MTT assay. Dashed line represents the no treatment control. Statistical analysis of cell viability in HCT116 (**B**), HeLa (**D**) and HFF (**F**) cells. Analysis was performed using no treatment control

As a first step towards determining the potential medicinal uses of extracts derived from Paeonia flowering plants, methanol extracts were obtained roots from five Paeonia species (*P. mascula* subsp. *mascula*, *P. daurica*, *P. mascula* subsp. *bodurii*, *P. arietina*, and *P. wittmanniana*) and tested for cytotoxic activity against three different types of human cells, including HCT116, HeLa, and Human Foreskin Fibroblasts (HFF) (Fig. 1, Supplementary Table 1). While HCT116 and HeLa cell lines are derived from human tumors, HFF cells are non-tumorigenic. No statistically significant change in cell viability was observed in cells treated at 5 µg/mL with any of the Paeonia-derived extracts in comparison to the untreated control (*p* > 0.05), as determined by two-way ANOVA followed by Dunnett’s multiple comparison test. Treatment of cells with higher concentrations of extracts resulted in decreased cell viability in all three cell types, with normal HFF cells more sensitive than the two tumor-derived cell lines. These results indicate that low concentrations of Paeonia-derived root extracts may be suitable for medicinal use.

As a starting point to determine if compounds present in Paeonia-derived root extracts are suitable for development as medicinal compounds, the Natural Product Activity and Species Source database (Zhao et al. 2023) (NPASS database, https://bidd.group/NPASS/) was used to identify 72 phytochemicals in Paeonia-derived extracts. These 72 compounds were first screened in silico to eliminate compounds with nuisance substructures or promiscuous scaffolds that are known to interfere with standard biochemical assays or form non-specific colloidal aggregates with proteins (Kaya and Colmenarejo 2020; Yang et al. 2016), yielding 31 compounds that were deemed suitable for further consideration. The Vina docking score of all 31 compounds is collected in Supplementary Table 2.

To assess the utility of AI-based computational tools for identifying Paeonia-derived phytochemicals with promising medicinal properties, we chose to target the main protease of SARS-CoV-2 as a proof-of-concept study. An in silico docking approach was used to identify Paeonia-derived compounds capable of binding to 3CL^pro^, the main protease of SARS-CoV-2. Multiple ligand-bound structures of 3CL^pro^ have been determined, including several structures that contain inhibitory compounds in the active site, including nirmatrelvir, the only FDA-approved 3CL^pro^ inhibitor (PDB file 8DZ2) (Noske et al. 2023), N3, a large peptide-based Michael acceptor molecule (PDB file 6LU7) (Jin et al. 2020) or the phytochemical baicalein (PDB file 6M2N) (Su et al. 2020). We used the baicalein-bound structure of 3CL^pro^ (6M2N) for initial docking of Paeonia-derived compounds, since all Paeonia-derived compounds used in this study are similar in size to baicalein, which is significantly smaller than either nirmatrelvir or N3. Baicalein and all other non-protein atoms were removed and missing sidechains, polar hydrogens and Kollman charges were added to 6M2N prior to docking. As a positive control for the docking experiments, baicalein was included along with the Paeonia-derived compounds.

**Fig. 2:**
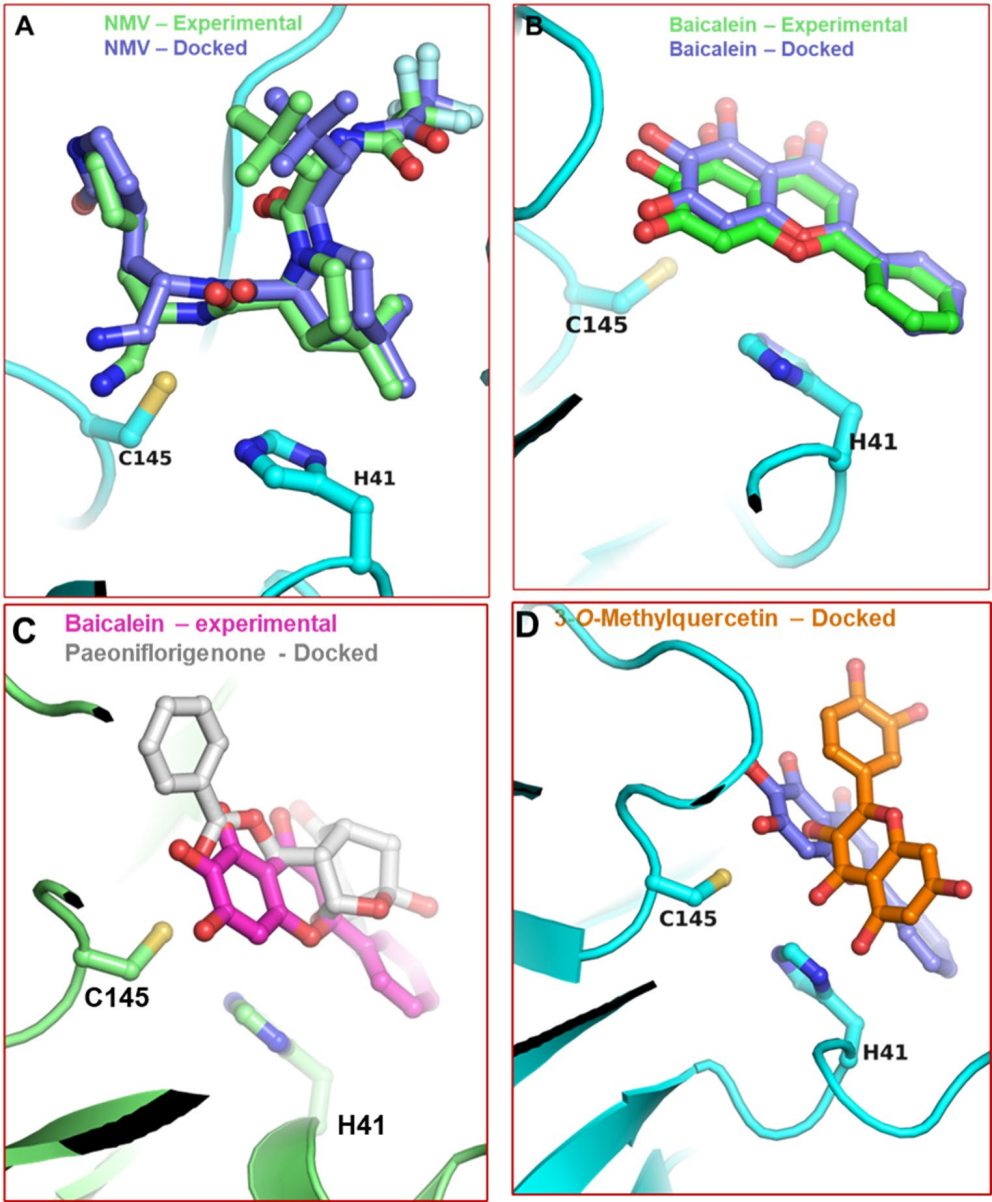
Validation of our docking protocol and details on interactions:** A** superposition of experimental (green carbons) and docked (purple carbons) nirmatrelvir (ball-and-stick) in the crystal structure of 3CLpro (PDB file 8DZ2).** B** superposition of experimental (green carbons) and docked (purple carbons) baicalein (ball-and-stick) in the crystal structure of 3CLpro (PDB file 6M2N).** C** superposition of paeoniflorigenone (docked; white carbons) and baicalein (experimental, purple) in the crystal structure of 3CLpro (PDB file 6M2N).** D** superposition of 3-O-methylquercetin (docked; orange carbons) and baicalein (experimental, purple) in the crystal structure of 3CLpro (PDB file 6M2N)

The 31 Paeonia-derived compounds, along with baicalein as a positive control, were used for parallel in silico docking experiments performed with AutoDock Vina (Eberhardt et al. 2021; Trott and Olson 2010) and with GNINA (McNutt et al. 2021, 2025). AutoDock Vina is a traditional molecular docking tool that relies on an empirical scoring function to estimate binding free energy, while GINNA employs convolutional neural networks (CNNs) to analyze protein-ligand interactions. Both AutoDock Vina and GNINA identified the same top 9 compounds, although the predicted rank order of binding affinity differed between the two methods (Table 1). All nine compounds were predicted to have a favorable binding energy of less than − 6.0 kcal/mol, comparable to the predicted binding energy of baicalein, done in parallel (Table 1). All nine compounds docked into the active site of SARS-CoV-2 3CL^pro^, overlapping with the experimentally determined position of baicalein. To validate our docking protocol, which used selected ML and/or DL tools, we first performed a benchmark analysis using nirmatrelvir and baicalein ligands in PDB files 8DZ2 and 6M2N, respectively. For this purpose, the ligands were removed from their respective PDB files prior to re-docking, followed by re-docking nirmatrelvir or baicalein into the active site of the ligand-free structure. For both nirmatrelvir and baicalein, the ligand poses obtained after redocking were in close agreement (RMSD < 0.5 Å) with the experimentally determined poses (Fig. 2A and B). The overlap of experimental NMV and docked NMV, experimental baicalein and docked baicalein, experimental baicalein and docked paeoniflorigenone, and experimental baicalein and docked 3-*O*-methylquercetin is shown in Fig. 2A, B and C, and 2D, respectively.

**Table 1:** Binding affinity (in kcal/mol) determined by AutoDock Vina and by GNINA

Ligands	Binding affinity (kcal/mol)	
Phytochemicals	VINA	GNINA
Paeoniflorigenone	− 7.4	− 8.02
Palbinone	− 7.5	− 7.98
Apigenin	− 7.5	− 7.97
Eriodictyol	− 7.1	− 7.91
Kaempferol	− 7.4	− 7.85
Cassythicine	− 7.7	− 7.49
Paeonilactone C	− 7.1	− 7.39
3-O-methylquercetin	− 6.7	− 7.39
Baicalein	− 6.7	− 6.89
(-)-Catechin	− 7.3	− 6.79

**Fig. 3:**
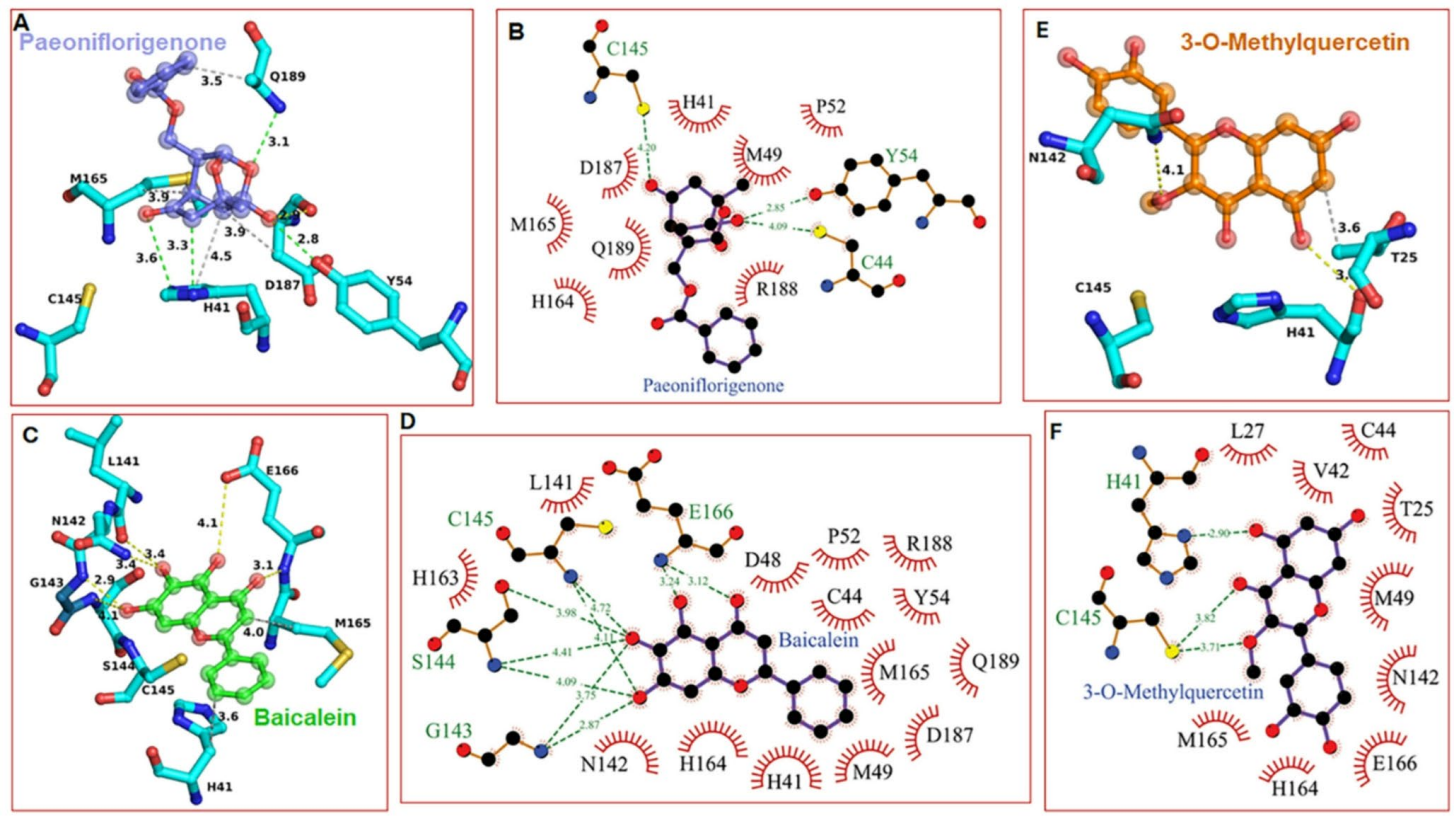
Detailed interactions of paeoniflorigenone, baicalein, and 3-O-methylquercetin within the SARS-CoV-2 main protease (3CLpro) active site.** A** Three-dimensional binding pose of paeoniflorigenone in the catalytic pocket of 3CL^pro^ (PDB file 6M2N), showing hydrogen bond interactions with key active-site residues including H41, C145, D187, Q189, M165, and Y54.** B** Two-dimensional interaction diagram of paeoniflorigenone highlighting hydrogen bonds (green dashed lines). The hydrophobic contacts (are shown as red arcs with the residues in the S1/S2 subsites.** C** Three-dimensional binding mode of baicalein demonstrating interactions with residues N142, G143, S144, C145, H41, E166, L141, and M165.** D** Two-dimensional interaction profile of baicalein, showing an extensive hydrogen-bonding network involving C145, H41, G143, S144, E166, and N142.** E** Three-dimensional binding pose of 3-O-methylquercetin within the catalytic site, illustrating interactions with H41, T25, N142, and C145.** F** Two-dimensional interaction map of 3-O-methylquercetin depicting hydrogen bonds and hydrophobic contacts with catalytic and surrounding residues, including H41, C145, M165, E166, T25, and V42. Green dashed lines represent hydrogen bonds; red semicircle arcs indicate hydrophobic interactions; bond distances are shown in angstroms (Å). The 2D interaction profile was generated with LigPlot+ (Laskowski & Swindells, 2011)

To gain insight into the details of the interactions, we analyzed the docking poses of paeoniflorigenone, baicalein, and 3-*O*-methylquercetin within the SARS-CoV-2 3CL^pro^, as shown in Fig. 2. These details are shown in Fig. 3. All three compounds docked close to the active site residues and interacted with nearby residues surrounding the catalytic dyad H41-C145. Paeoniflorigenone formed hydrogen bonds with Q189 and D187. Baicalein displayed an extended hydrogen-bonding network involving N142, G143, S144, and E166. 3-*O*-methylquercetin also showed strong interactions with the residues near the catalytic site justifying its efficacy comparable to baicalein. Collectively, these models support direct active-site binding as the structural basis for inhibition.

To further validate our docking protocol, which used selected ML and/or DL tools, we first performed a benchmark analysis using nirmatrelvir and baicalein ligands in PDB files 8DZ2 and 6M2N, respectively. For this purpose, the ligands were deleted from their respective PDB files prior to re-docking, followed by re-docking of nirmatrelvir or baicalein into the active site of the respective ligand-free structure. For both nirmatrelvir and baicalein, the ligand pose obtained after re-docking was in close agreement (RMSD less than 0.5 angstrom) with the experimentally determined pose (Fig. 2).

**Fig. 4:**
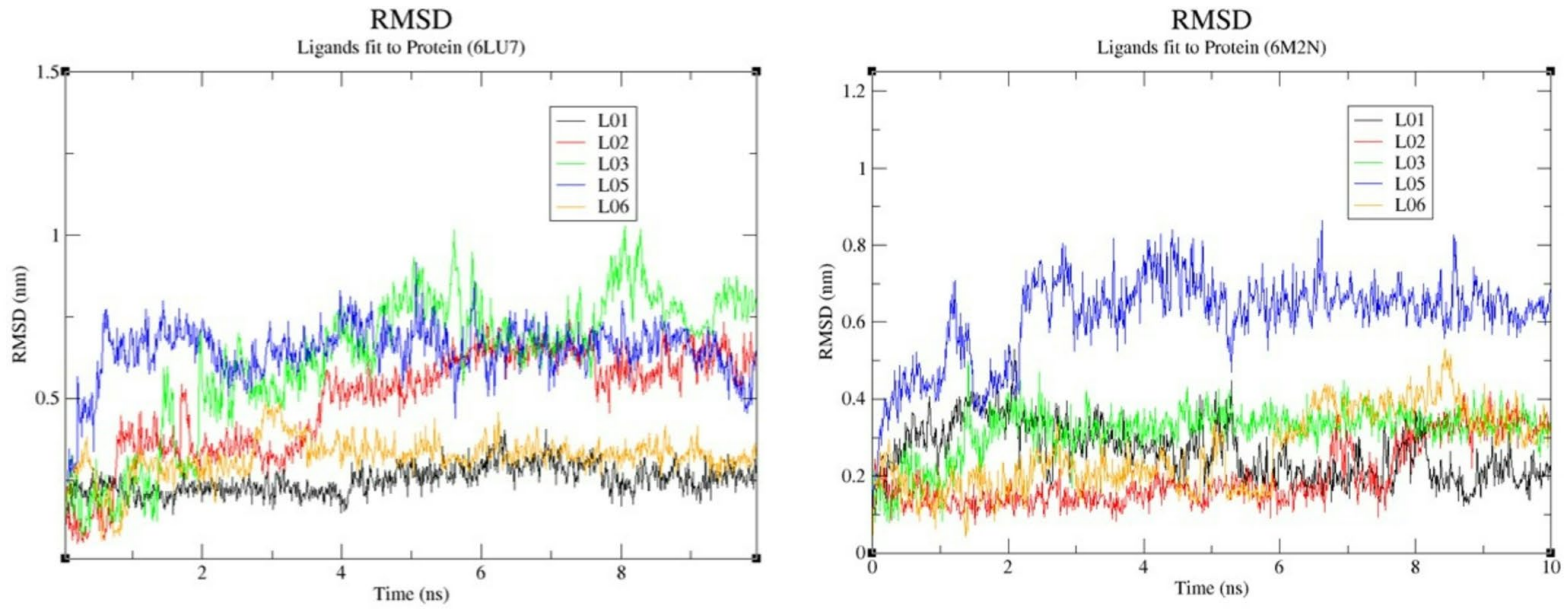
RMSD analysis plot of ligands docked into 6LU7 over a 10 ns MD simulation. Ligands are (-)-cathecin, apigenin, palbinone, paeoniflorigenone, and eriadictyol

**Fig. 5:**
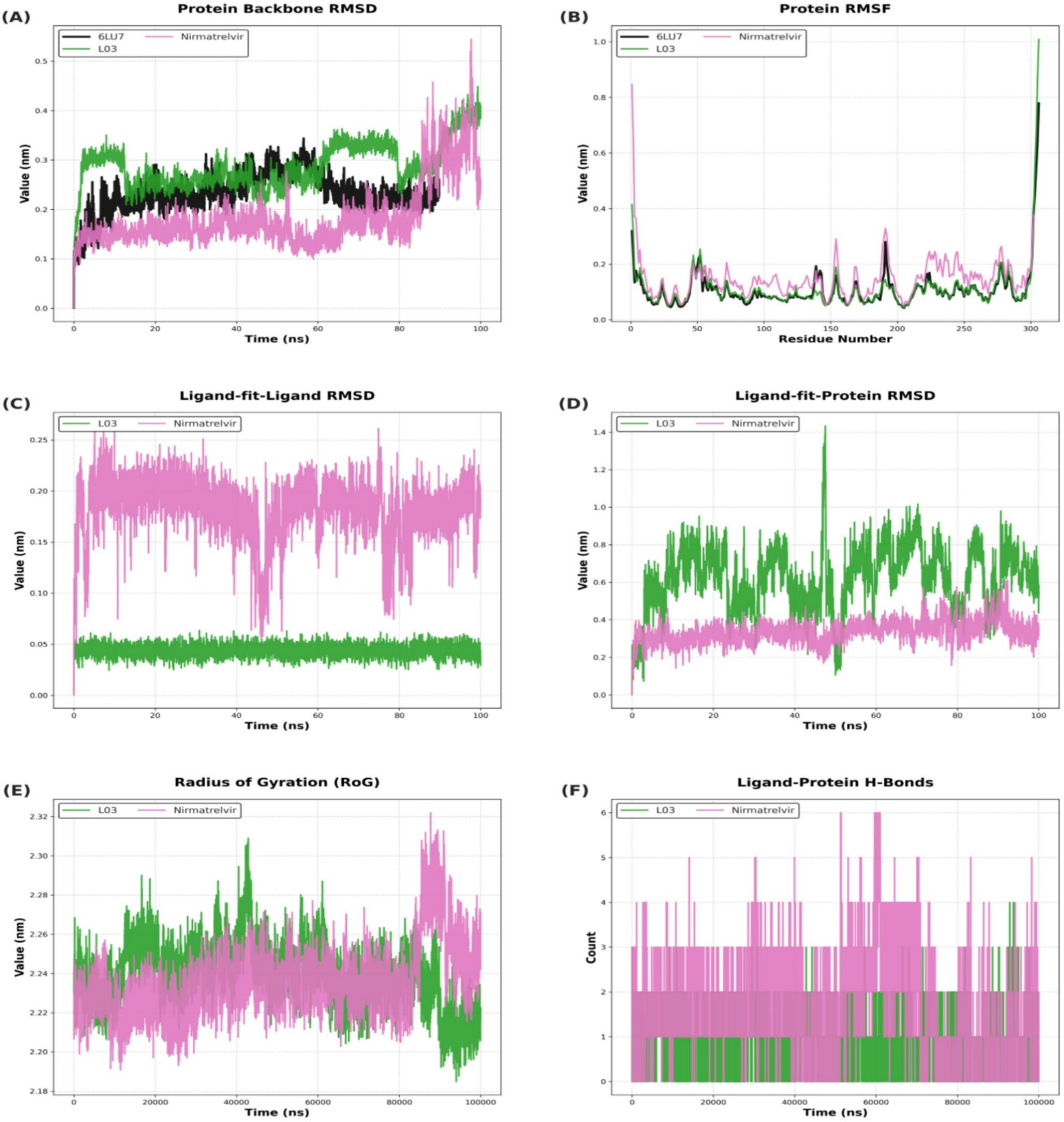
Comparative MD Analysis of 6LU7 Main Protease in Apo and Ligand-Bound States. This figure illustrates the structural stability and dynamic behavior of the 6LU7 protein throughout a 100 ns simulation: the apo form (6LU7, black) and 2 ligand-bound complexes (palbinone (L03 - 6LU7, green) and nirmatrelvir (8DZ2, pink).** A** Protein Backbone RMSD,** B** Protein RMSF,** C** Ligand-on-Ligand RMSD,** D** Ligand-on-Protein RMSD,** E** Radius of Gyration (RoG),** F** Ligand-Protein H-Bonds

**Fig. 6:**
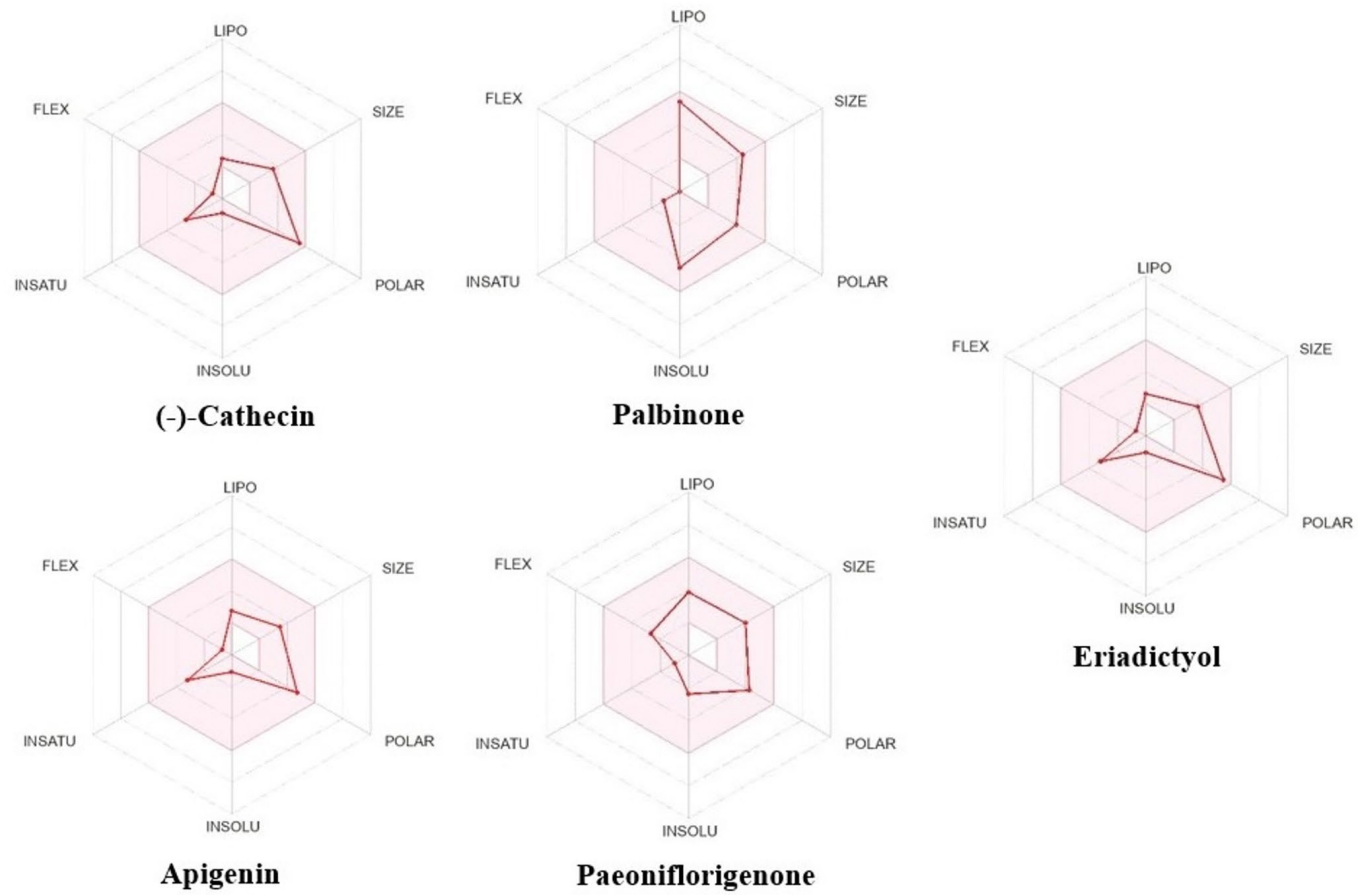
ADME diagrams showing lipophilicity (LIPO), molecular mass (SIZE), polarity, (POLAR), solubility (INSOL), carbon-carbon double bonds (INSATU) and flexibility (FLEX)

To evaluate the binding stability of candidate ligands to SARS-CoV-2 3CL^pro^, five of the top-ranked compounds were analyzed for ligand RMSD trajectories from molecular dynamics (MD) simulations using both 6M2N and 6LU7 (Fig. 4). In the 6M2N structure, four compounds, including (-)-catechin, apigenin, palbinone and eriadictyol, had RMSD values between 0.1 and 0.4 nm, while the RMSD value of paeoniflorigenone was 0.6 and 0.8 nm, suggesting decreased stability for the paeoniflorigenone-containing complex. In the 6LU7 structure, only (-)-catechin) and eriadictyol had RMSD values less than 0.5 nm while palbinone, eriadictyol and paeoniflorigenone had RMSD values greater than 0.6 nm. To identify the most promising inhibitors for the 6LU7 protease, an initial 100 ns Molecular Dynamics (MD) simulation was performed across various protein-ligand systems. Compounds such as L01, L02, L05, and L06 exhibited high positional drift, often exceeding 2.0 nm, indicating a lack of stable occupancy in the catalytic pocket. These systems were consequently eliminated. Following the elimination process, nirmatrelvir and palbinone (L03) were identified as the primary leads (Fig. 5B). The protein backbone RMSD (Fig. 5A) indicates that the systems reached a stable plateau after approximately 20 ns. Nirmatrelvir (pink) exhibited a notably lower average RMSD (~ 0.15 nm) compared to the apo (black) and L03 (green) systems for the majority of the trajectory. However, a sharp increase in backbone RMSD for the nirmatrelvir complex was observed after 85 ns, peaking at approximately 0.5 nm. The Root Mean Square Fluctuation (RMSF) analysis (Fig. 5B) shows the flexibility of individual amino acid residues. Most secondary structure elements remained rigid with fluctuations below 0.2 nm. Ligand-on-Ligand (Fig. 5B-C), L03 showed minimal internal conformational changes (~ 0.05 nm). In contrast, nirmatrelvir exhibited higher internal fluctuations (~ 0.2 nm), indicating a more flexible molecular structure. Ligand-on-Protein (Fig. 5C), nirmatrelvir demonstrated superior binding stability within the catalytic pocket, maintaining a position around 0.3–0.4 nm. L03 showed significant positional drift, with values fluctuating between 0.6 nm and 1.0 nm, and a sharp spike reaching 1.4 nm at 45 ns. The Radius of Gyration (RoG) (Fig. 5E) remained stable between 2.22 and 2.28 nm for both complexes. Nirmatrelvir showed a slight expansion in RoG during the final 15 ns, correlating with its backbone RMSD increase. Regarding intermolecular interactions, nirmatrelvir formed a more extensive hydrogen bond network (Fig. 5F), consistently maintaining 2–4 bonds and peaking at 6, whereas L03 frequently dropped to 0–1 bonds throughout the simulation. Nirmatrelvir maintained a remarkably low backbone RMSD (~ 0.15 nm) for the majority of the 100 ns run. While L03 showed a higher deviation (~ 0.3 nm) than nirmatrelvir, it remained significantly more stable than the previously eliminated compounds, justifying its inclusion in the detailed comparative analysis. To assess if the Paeonia-derived compounds had physicochemical properties compatible with oral bioavailability, the ADME properties for five of the Paeonia-derived compounds, including catechin, palbinone, apigenin, paeoniflorigenone, and eriadictyol, were calculated using SwissADME (Daina et al. 2017). The Radar plots from SwissADME are shown in Fig. 6. All compounds were within the desired physicochemical property range, indicative of likely favorable oral bioavailability. To prioritize compounds for further evaluation, we defined a composite Lead Score (LS) that integrates binding affinity, predicted toxicity, synthetic accessibility, and lipophilicity of the compounds to predict compounds with favorable drug-like properties (Table 2). Lead Scores for ten compounds, including baicalein, are provided in Table 2, along with Lipinski’s rule of 5 and PAINS (Pan Assay Interference). Three compounds (paeoniflorigenone, paeonilactone C and cassythicine) were predicted to have drug-like properties as well as high binding affinity to SARS-CoV-2 3CL^pro^ protease. According to the pkCSM-based ADME analysis in Table 3, compounds with high Caco-2 permeability (predicted values > 0.90), intestinal absorption exceeding 30%, and log pK values greater than − 2.5 were deemed to have favorable pharmacokinetic properties, supporting their selection for further molecular docking and drug-likeness evaluation.

**Table 2:** Calculation of lead scores for Paeonia-derived compounds

Compounds	Binding affinity (kcal/mol)	Tox		(SA_Raw_)		LogP		Lead score		Ro5	PAINS			
Paeoniflorigenone	− 8.02	0.54		1.16		1.27		12.74		NO	NO			
Eriodictyol	− 7.91	0.63		2.23		2.22		12.4		NO	YES			
Paeonilactone C	− 7.98	0.58		1.59		1.12		12.33		NO	NO			
Cassythicine	− 7.49	0.5		1.95		2.88		12.32		NO	NO			
Apigenin	− 7.97	0.67		3.57		2.58		11.81		NO	NO			
Kaempferol	− 7.85	0.65		3.28		2.28		11.8		NO	NO			
3-O-methylquercetin	− 7.39	0.61		3.02		2.29		11.56		NO	YES			
(-)-Catechin	− 6.79	0.65		1.86		1.55		11.08		NO	YES			
Baicalein	− 6.89	0.64		3.58		2.58		10.78		NO	YES			
Palbione	− 7.82	0.24		4.63		3.05		10.75		NO	YES			

Baicalein was included in our docking as it is bound in the crystal structure of 3CL^pro^ (PDB file 6M2N)

The Lead Score (LS) was calculated as described in Eq. 1 (see “Materials and methods”). Binding affinity, or − ΔGdock, represents the GNINA-predicted convoluted neural network (CNN) binding affinity. Tox is the eToxPred-predicted toxicity score (0–1, with 0.58 considered to be discriminatory between toxic and non-toxic molecules (Pu et al. 2019). SA_raw_ is the raw synthetic accessibility score (1–10) (Pu et al. 2019). LogP is the RDKit-predicted partition coefficient (Wildman and Crippen 1999). Compounds with higher Lead Scores are predicted to be more potent, safer, synthetically feasible, and have favorable lipophilicity. Ro5 indicates if the compound satisfies Lipinski’s Rule of 5 (Lipinski 2004) and PAINS indicates if the compound is likely to have Pan Assay Interference properties (Baell and Nissink 2018)

**Table 3:** Predicted pharmacokinetic properties of the selected compounds generated using the pkCSM server

Compounds	Water solubility (log mol/L)		Caco2 permeability (log Papp in 10–6 cm/s)	Intestinal absorption (human) (% Absorbed)	Skin permeability (log Kp)	*P*−glycoprotein substrate	*P*−glycoprotein I inhibitor	*P*−glycoprotein II inhibitor	H bond acceptors	H bond donors
(−)−Catechin	− 3.124		− 0.17	69.21	− 2.735	Yes	No	No	6	5
Apigenin	− 3.38		1.062	91.566	− 2.744	Yes	No	No	1	5
Palbinone	− 4.551		1.078	94.214	− 4.397	Yes	Yes	No	4	2
Kaempferol	− 3.282		0.195	80.064	− 2.735	Yes	No	No	6	4
Paeoniflorigenone	− 2.944		0.09	75.123	− 3.566	No	No	No	6	1
Eriodictyol	− 3.041		0.359	75.138	− 2.748	Yes	No	No	6	4
PaeonilactoneC	− 3.22		0.196	69.713	− 3.162	No	No	No	6	1
Cassythicine	− 2.752		1.204	93.58	− 2.752	Yes	No	Yes	5	1
3−o−methylquercetin	− 3.249		− 0.196	87.457	− 2.735	Yes	No	No	7	4

High Caco-2 permeability was indicated by predicted values > 0.90, along with intestinal absorption

**Fig. 7:**
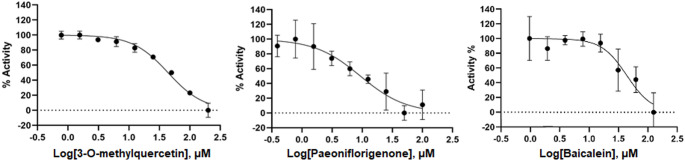
Inhibition of SARS-CoV-2 3CLpro activity by 3-O-methyl-quercetin, paeoniflorigenone and baicalein. The IC50 value for 3-O-methyl-quercetin, paeoniflorigenone and baicalein were 43.94, 9.33 and 43,85 µM respectively. The enzyme was incubated with the compounds at the indicated concentrations. Enzyme activity was measured by quantification of ﬂuorescence released from the quenched peptide following exposure to the protease

To evaluate the results of our in silico approach, we measured the ability of paeoniflorigenone, the top-ranked compound, and 3-*O*-methylquercetin, a mid-ranked compound, to inhibit the SARS-CoV-2 3CL^pro^ protein. We used baicalein as a control. The SARS-CoV-2 3CL^pro^ protein was expressed and purified from bacteria as a glutathione-S-transferase (GST) fusion protein. Following removal of the GST tag with Factor Xa, the activity of the purified SARS-CoV-2 3CL^pro^ was measured using the fluorogenic substrate Ac-Abu-Tle-Leu-Gln-AFC (Rut et al. 2021). The K_m_ value of the substrate for the protease was determined to be 16 µM. To determine the ability of 3-*O*-methylquercetin, paeoniflorigenone, and baicalein to inhibit the protease, the enzyme was incubated with serial dilutions of the compounds prior to the addition of the substrate at 60 µM. An excess of substrate, approximately 4-fold greater than the Km, ensured that substrate did not become limiting during the time course of the reaction. After the addition of substrate, product formation was continuously monitored for 5 min using a plate reader, and IC_50_ values were determined by nonlinear regression. The IC_50_ values for 3-*O*-methylquercetin, paeoniflorigenone and baicalein were 43.94 µM, 9.33 µM and 43,85 µM respectively (Fig. 7).

## Discussion

The purpose of this proof-of-concept study was to evaluate the ability of AI-based computational tools to identify Paeonia-derived phytochemicals with promising utility as medicinal compounds. To this end, our approach integrated traditional and AI-based docking tools, AI-based drug-likeliness prediction as well as experimental validation of inhibition of SARS-CoV-2 3CL^pro^ protease. Our results demonstrate that paeoniflorigenone inhibits the SARS-CoV-2 3CL^pro^ protease with an IC_50_ in the low micromolar range, lower than that of baicalein. Prior characterization of the biological activities of paeoniflorigenone has focused on its anti-inflammatory and anti-tumor activities, as well as its ability to inhibit both bacterial and viral DNA polymerases (Demir et al. 2019; Huang et al. 2017; Li et al. 2021). Our study is the first to show that paeoniflorigenone is a strong candidate for further development as an inhibitor of the SARS-CoV-2 3CL^pro^.

A previous study has demonstrated that Shuanghuanglian, a traditional Chinese medicine used for the treatment of acute respiratory infections, contains compounds capable of inhibiting SARS-CoV-2 3CL^pro^ protease activity (Su et al. 2020). The principal inhibitory compound in the Shuanghuanglian preparations was shown to be baicalin, a glycoside derivative of baicalein (5, 6, 7-tryhydroxyflavone). Both baicalin and baicalein bound to SARS-CoV-2 3CL^pro^, with K_d_’s of 11.5 µM and 4 μm, respectively (Su et al. 2020). A crystal structure of baicalein bound at the active site of SARS-CoV-2 3CL^pro^ (PDB 6M2N) indicated close contacts of baicalein with multiple residues in the active site, including the catalytic dyad of Cys145 and His41 (Su et al. 2020). Our docking experiments indicate that the Paeonia-derived compounds examined in this study bind in the SARS-CoV-2 3CL^pro^ active site in a manner similar to that of baicalein. For example, the paeoniflorigenone-binding site in SARS-CoV-2 3CL^pro^ has significant overlap with the binding site of baicalein, with close contacts between paeoniflorigenone and the catalytic dyad (Fig. 2C). Consistent with a highly similar mode of binding, baicalein and paeoniflorigenone have similar inhibitory activity against SARS-CoV-2 3CL^pro^. Notably, when other drug-like properties are considered, including potential toxicity and PAINS, we suggest that paeoniflorigenone may be the preferred compound for further drug development.

It should be pointed out here that the 3-*O*-methylquercetin and baicalein share a common flavonoid scaffold. However, the substituted chemical groups differ between the two molecules, which can potentially influence binding orientations within the 3CL protease active site. Docking with GNINA suggests that the C3-methyl group in 3-*O*-methylquercetin may not favor the planar orientation observed for baicalein due to steric constraints near residues such as Met165 and Glu166. In contrast, the catechol (3′,4′-dihydroxy) moiety on the B-ring of 3-*O*-methylquercetin can interact with residues such as H163 within the S1 subsite (Fig. 3F). These interactions are not available to baicalein due to its distinct OH groups (Fig. 3E). Such differences in substituent topology are likely to orient ligands differently within the binding pocket, resulting in distinct predicted poses despite a shared core scaffold. Since these binding poses are derived from GNINA docking, they should be interpreted as plausible binding modes instead of experimental results.

The pkCSM predictions for the nine compounds evaluated in the study show distinct differences in terms of oral bioavailability; although all compounds exhibit human intestinal absorption above 30%, only apigenin, palbinone, and cassythicine surpass the high Caco-2 permeability threshold, indicating strong epithelial transport potential, whereas (−)-catechin and 3‑*O*‑methylquercetin stand out with low permeability values.

The initial 100 ns MD production runs served as a critical filtration step in our computational pipeline. The elimination of several ligands (L01, L02, L05, and L06) was necessitated by their high Ligand-on-Protein RMSD values. In MD studies, a ligand drift exceeding a specific threshold (typically > 0.5 nm in a confined pocket) is a strong indicator of weak binding affinity or potential dissociation. The failure of these ligands to stabilize the 6LU7 backbone suggests they are unable to overcome the conformational strain of the active site.

The exclusion of these systems allowed for a focused discussion on the molecular mechanisms of nirmatrelvir and palbinone (L03**)**. The high internal flexibility of nirmatrelvir (Lig-on-Lig RMSD, Fig. 5B-C) coupled with its superior binding stability (Lig-on-Prot RMSD, Fig. 5B-D) suggests an “induced fit” mechanism. This flexibility allows nirmatrelvir to optimize its geometry to maximize van der Waals and electrostatic contacts with the catalytic residues, a feature notably absent in the eliminated, more rigid, yet poorly bound ligands. It should be noted that nirmatrelvir acts as a covalent inhibitor in experimental settings. However, in our current MD setup, it was modeled as a non-covalent binder. It is possible that the selection of this method may lead to an overestimation of the conformational freedom and RMSD values of the ligand. Therefore, the observed flexibility of nirmatrelvir in our simulations should be interpreted as its intrinsic behavior within the pocket prior to or in the absence of covalent bond formation.

In conclusion, the 100 ns MD screening successfully narrowed the candidate pool by filtering out ligands with unstable trajectories and weak hydrogen bonding profiles. This robust selection process ensures that only compounds with high structural persistence and significant interaction networks, like palbinone (L03), are considered for final lead optimization.

## Conclusion

In summary, this study demonstrates that a combination of traditional and AI-based computational tools can be effectively used to identify phytochemicals, which can subsequently be validated and optimized through in vitro and in vivo assays against defined antiviral targets. Using Paeonia-derived phytochemicals as examples, we identified potential anti-SARS-CoV-2 phytochemicals targeting the viral main 3CL^pro^. A key limitation of the study is the relatively low to moderate efficacy of these phytochemicals against 3CL^pro^. However, modern cheminformatics platforms that explore vast chemical spaces can be integrated into lead optimization pipelines to refine initial phytochemical scaffolds, potentially yielding more potent antiviral agents.

## Materials and methods

### Paeonia taxa and compound extraction

 Five taxa of Paeonia genus, *P. mascula* subsp. *mascula*, *P. daurica*, *P. mascula* subsp. *bodurii*, *P. arietina*, *P. wittmanniana*, were collected from different provinces in Turkiye (Afyonkarahisar, Mersin, Çanakkale, Gümüşhane and Rize), respectively. The powdered root (10 g) was added to 50 mL of 100% methanol and kept at 37 °C on a shaker at a controlled speed for 2.5 h (Heidolph Unimax 1010, Germany). Ultrasonic (Elma Clean Box, Elma) extraction was applied at 25 °C for one hour, followed by centrifugation 4,000 rpm for 20 min at 4 °C. The supernatants were collected and evaporated using EZ-2 Evaporator (GeneVac, Gardiner, NY).

### Cell culture assays

The cytotoxic effect of five plant extracts was assessed in three cell types: HCT116 (human colorectal carcinoma), HeLa (human cervical carcinoma), and HFF (human foreskin fibroblast). The cells were maintained in humidified conditions and incubated at 37 °C with 5% CO_2_. The cells were cultured in DMEM culture media, supplemented with 10% heat-inactivated fetal bovine serum, 1% Pen Strep, and 1% MEM-NEAA. All cell culture reagents were purchased from ThermoFisher Scientific (Waltham, MA). For the MTT cell viability assay, 20,000 cells/well were plated in 96-well plates. Cells were then treated with different plant extract samples at 5, 20, 45, and 80 µg/ml for 48 h. After that, MTT solution was added to each well and incubated at 37 °C for 3 h, followed by solubilization. Absorbance was measured at 570 nm using the SYNERGY-Neo2 BioTek plate reader. The results were analyzed using the following equation:

1 $$\:Cell\:Viability\:\left(\%\right)=\:\frac{Absorbance\:of\:treated\:well}{Absorbance\:of\:non-treated\:negative\:control}\:\times\:100$$

All MTT cell viability assays were performed in quadruplicate (*n* = 4). Statistical analysis was performed using two-way ANOVA followed by Dunnett’s multiple comparison test. Statistical analysis was performed using Prism 9.5.1. software (GraphPad, Boston, MA). Error bars ± standard error of the mean (SEM).

### Collection and preparation of protein structures

Three PDB files of SARS-CoV-2 3CL^pro^, each containing a different ligand in the active site of 3CL^pro^, including 8DZ2 (Noske et al. 2023), 6LU7 (Jin et al. 2020) and 6M2N (Su et al. 2020) were downloaded from https://www.rcsb.org. The PDB files were processed through OpenBabel (O’Boyle et al. 2011) and the MDAnalysis Python library (Michaud-Agrawal et al. 2011) to generate PDBQT files, in which all water molecules and other heteroatoms (HETATMs) were removed and missing sidechains, polar hydrogens and Kollman charges were added. Potential binding pockets and sub-pockets in the protein structures were detected by DockSiteScorer (Volkamer et al. 2012) and P2Rank (Krivak and Hoksza 2018), an ML-based tool for predicting ligand binding sites in protein structures. For both DoGSiteScorer and P2Rank, the top-ranked (highest score) location of the predicted binding pocket for baicalein in 6M2N and nirmatrelvir in 8DZ2 was the binding pocket reported in the respective crystal structures (Noske et al. 2023; Su et al. 2020). All ligands from the NPSS database were docked in this pocket, which is at the active site of 3CL^pro^. In the docking experiments, the docking exhaustiveness parameter was set to 8. The docking box center and grid size were computed using the Bio.PDB module of the Biopython library (Cock et al. 2009), using an in-house Python script.

### Selection of phytochemicals and ligand preparation

 The NPASS database (https://bidd.group/NPASS/)(Zhao et al., 2023) contains 72 phytochemicals known as Paeonia-derived compounds. For all 72 compounds, either 3D-SDF (3D-Structure Data Format) or SMILES (Simplified Molecular Input Line Entry System) coordinates were downloaded from the PubChem database (Kim et al. 2023) (Supplementary Table 2). Ligand structures formatted as SMILES strings were processed using a custom in-house Python pipeline that used OpenBabel (O’Boyle et al. 2011) to convert SMILES to 3D-SDF format. Before docking experiments were undertaken, the 72 Paeonia-derived compounds were pre-screened to eliminate compounds containing nuisance substructures, promiscuous scaffolds or that have a propensity to form non-specific colloidal aggregates with proteins (Kaya and Colmenarejo 2020; Yang et al. 2016). This screening process eliminated 41 compounds, leaving 31 compounds whose structures were optimized using the MMFF94 force field (Halgren 1996) prior to docking. To identify the most promising leads for experimental validation, we applied an affinity-based prioritization strategy based on consensus docking scores and structural complementarity within the 3CL^pro^ active site. Only the top 9 compounds, candidates that met or exceeded the predicted binding affinity of the positive control (baicalein), were selected for detailed analysis. An in-house Python pipeline (available upon request) was used to generate PDBQT files of all ligand molecules that were used for docking.

### Docking

AutoDock Vina (Eberhardt et al. 2021; Trott and Olson 2010) and GNINA (McNutt et al. 2021, 2025) were used to predict binding affinities of the Paeonia compounds with SARS-CoV-2 3CL^pro^. AutoDock Vina represents a traditional molecular docking tool that relies on an empirical scoring function to estimate binding free energy. In contrast, GNINA is an advanced DL-based docking technique that employs convolutional neural networks (CNNs) to analyze protein-ligand interactions and affinity. We selected GNINA as the deep learning-based docking tool because it employs a three-dimensional convolutional neural network (CNN) to evaluate spatial and chemical properties of amino acids and ligands at the protein/ligand interface. It was used to complement the empirical scoring function of AutoDock Vina by providing a CNN-based estimate of binding affinity. This approach previously validated for improved pose prediction and ligand ranking performance. The composite Lead Score (LS) was used as the final prioritization metric, integrating docking affinity estimates and developability-related predictions into a single heuristic score to guide compound selection.

First, AutoDock Vina (through PyRx (Dallakyan and Olson 2015) was used to rank order the 31 Paeonia-derived compounds based on their predicted binding affinity for 3CL^pro^. Following this primary screen, the compounds were prioritized using a dual-scoring strategy. The compounds with only those compounds demonstrating a high consensus affinity (GNINA score ≤ -6.89 kcal/mol) were subjected to further in-depth evaluation. Nine compounds with the highest binding affinity as determined by AutoDock Vina were then docked using GNINA, incorporating an in-house Python script using appropriate receptors and ligands. Docking grid center and grid size were determined using Bio.PDB library through an in-house Python script. After docking, visual assessment of the poses with the lowest binding energies, in kcal/mol, was carried out in PyMOL (The PyMOL Molecular Graphics System, Version 2.5, Schrödinger, LLC).

### Lead score

To prioritize top-ranked candidate compounds for further evaluation, we defined a composite Lead Score (LS) that integrates binding affinity, predicted toxicity, synthetic accessibility, and lipophilicity of the compounds. This heuristic scoring function was designed for rewarding strong binders with low toxicity, ease of synthesis, and optimal lipophilicity. Compounds with higher LS Scores are predicted to be more potent, safer, synthetically feasible, and have favorable lipophilicity.

The LS formula is:

2 $$ \begin{aligned} LS = & - \Delta \:G_{{dock}} + 2 \times \:\left( {1 - Tox} \right) + 0.5 \times \:\left( {10 - SA_{{raw}} } \right) \\ & - 0.5 \times \:\left| {LogP - 2.5} \right| \\ \end{aligned} $$

where ΔG_dock_ is the GNINA-predicted binding affinity (CNN affinity expressed in kcal/mol), Tox is the predicted toxicity score (0–1, where lower is safer), SA_raw_ is the raw synthetic accessibility score (1–10, lower score means easier synthetic accessibility), and LogP is the partition coefficient (Wildman and Crippen 1999). Tox and SA_Raw scores were predicted using eToxPred, an ML-based approach (Pu et al. 2019). LogP was predicted using RDkit Python library, https://www.rdkit.org. Compliance with Lipinski’s Rule of Five was evaluated using standard physicochemical descriptors implemented in RDKit, including molecular weight, cLogP, hydrogen bond donors, and hydrogen bond acceptors. Potential pan-assay interference compounds (PAINS) were identified using the RDKit FilterCatalog module, which implements the PAINS A, B, and C SMARTS patterns as originally described by Baell and Holloway (2010).

In silico ADME and pharmacokinetic analyses were performed using the pkCSM prediction platform. The evaluated parameters included aqueous solubility, Caco-2 permeability, human intestinal absorption, skin permeability (log Kp), P-glycoprotein substrate and inhibitor status, and hydrogen bonding capacity. Compounds meeting the criteria of high Caco-2 permeability (> 0.90), intestinal absorption above 30%, and log Kp values greater than − 2.5 were regarded as having favorable pharmacokinetic profiles and were advanced for subsequent molecular docking and drug-likeness analyses.

### Molecular dynamics simulation

 Molecular dynamics (MD) simulations using GROMACS (v2023.2) (Abraham et al. 2015; Valdes-Tresanco et al. 2021) and CHARMM36 force field (ff) (Vanommeslaeghe et al. 2010) were used to evaluate changes in protein conformation upon ligand binding. Water molecules (TIP3P) were added, and the protein-ligand complexes (Paeonia ligands − 6LU7 and nirmatrelvir − 8DZ2) were solvated in 150 mM NaCl at 310 K. A triclinic simulation cell, 10 Å larger than the protein, was used. The Particle Mesh Ewald method was applied to calculate long-range electrostatic interactions, and the modified Berendsen thermostat (V-rescale) was used to control the temperature throughout the simulations. The MD simulation time was set to 10 ns and 100 ns at 310 K temperature. The simulation results were analyzed using the default script of gmx rms to obtain root mean square deviation (RMSD), and gmx rmsf to obtain root mean square fluctuation (RMSF). Hydrogen bonds (H-bonds) and Radius of Gyration were analyzed by the molecular visualization program VMD (Humphrey et al. 1996).

### Efficacy of paeoniflorigenone and 3-O-methylquercetin against SARS-CoV-2 3CL^pro^

 The expression vector pGEX-5X-3-SARS-CoV-2-3CL^pro^ was obtained from Addgene (catalog #168457). The expression and purification of SARS-CoV-2 3CL^pro^ were performed as previously described (Iketani et al. 2021) with minor modifications. The N-terminal GST-tag of purified SARS-CoV-2 3CL^pro^ was removed by Factor Xa (New England/BioLabs, Ipswich, MA, USA) cleavage. The activity of SARS-CoV-2 3CL^pro^ was measured as previously described (Rut et al. 2021), using the fluorogenic substrate Ac-Abu-Tle-Leu-Gln-AFC (Sigma-Aldrich, Saint Louis, MO). Different concentrations of the substrate (ranging between 5 and 100 µM) were prepared in a buffer (50 mM Tris-HCl, pH 7.5) in a 96-well plate format. Purified 3CL^pro^ was then added to each well to a final concentration of 0.3 µM, followed by measurement of fluorescence (excitation wavelength 380 nm, emission wavelength 500 nm) on a plate reader (Millipore-Sigma, Milwaukee-Wisconsin) for 10 min. A K_m_ of 16 µM of the substrate for the protease was calculated by nonlinear regression (GraphPad Prism, Boston, MA). Serial dilutions of 3-O-methylquercetin (200 µM to 0.39 µM) (Millipore Sigma, Milwaukee, WI, USA), paeoniflorigenone (100 µM to 0.39 µM) and baicalein (125 µM to 0.97 µM) (MedChemExpress, Monmouth Junction, NJ, USA) were prepared in assay buffer and incubated with 0.3 µM 3CL^pro^ for 10 min at 37 °C. The substrate was then added at 60 µM per well, and fluorescence (excitation wavelength 380 nm, emission wavelength 500 nm) was continuously measured on a plate reader for 5 min. Inhibition was then calculated by comparison to control wells without inhibitor or enzyme. IC_50_ values were determined by nonlinear regression (GraphPad Prism, Boston, MA).

## Supplementary Information

Supplementary Material 1

## Data Availability

The authors confirm that the Data used and/or analyzed in the present study are available within the article.

## Abbreviations

nsp16: 2´-O-ribose methyltransferase
3CL^pro^ or M^pro^: main protease, or M^pro^
CNNs: Convolutional Neural Networks
COVID-19: Coronavirus disease 19
DL: Deep Learning
nsp15: Non-structural protein 15 (Endoribonuclease)
E: Envelope
GST: Glutathione-S-transferase
nsp10: Non-structural protein 10 (a cofactor of nsp14)
HFF: Human Foreskin fibroblasts
LS: Lead Score
M: Membrane
MERS-CoV: Middle East Respiratory Syndrome Coronavirus
MD: Molecular dynamics
NPASS: Natural Product Activity and Species Source
N: Nucleocapsid
ORFs: Open Reading Frames
PL^pro^: Papain-like protease
nsp14: Non-structural protein 14 (exoribonuclease)
SARS-CoV-2: Severe Acute Respiratory Syndrome Coronavirus 2
nsp9: Non-structural protein 9 (single-stranded RNA binding protein)
S: Spike
nsp12: Non-structural protein 12 (viral RNA-dependent RNA polymerase)
