# Supplementary material for: Targeting SARS-CoV-2 main protease (3CLpro) with Paeonia-derived phytochemicals
Source: In Silico Pharmacol. 2026 Apr 17;14(2):122. doi: 10.1007/s40203-026-00630-7 (PMC13090440; doi:10.1007/s40203-026-00630-7)
Supplement: Supplementary file 1 — Supplementary Material 1 [file 40203_2026_630_MOESM1_ESM.docx]

**Supplementary Table 1. EC_50_ for the samples 1-5 on HCT116, HeLa, and HFF**

| Treatment Group | *EC*_50_ (µg/ml) | | |
| --- | --- | --- | --- |
|  | HCT116 | HeLa | HFF |
| *P. daurica* | 85.69 | 75.60 | 59.46 |
| *P. mascula mascula* | 101.70 | 104.15 | 65.08 |
| *P. mascula bodurii* | 109.30 | 94.39 | 77.97 |
| *P. arietina* | 174.55 | 177.65 | 105.38 |
| *P. witmanniana* | 78.70 | 84.17 | 53.09 |

**Supplementary Table 2: Vina docking score of all 31 compounds**

| **Compound** | **Vina Score kcal/mol** |
| --- | --- |
| Cassythicine | -7.732 |
| Apigenin | -7.512 |
| Palbinone | -7.458 |
| Kaempferol | -7.432 |
| Paeoniflorigenone | -7.416 |
| (-)-Catechin | -7.341 |
| NPC261084 | -7.155 |
| Eriodictyol | -7.112 |
| Paeonilactone C | -7.092 |
| N-(4-hydroxyphenethyl)cinnamamide | -7.08 |
| NPC318613 | -7.043 |
| Hyoscyamine | -6.819 |
| Paeonidangenin | -6.819 |
| NPC79943 | -6.763 |
| NPC53763 | -6.752 |
| 3-O-methylquercetin | -6.742 |
| Oxyresveratrol | -6.727 |
| Baicalein | -6.714 |
| NPC107183 | -6.472 |
| Resveratrol | -6.46 |
| NPC48645 | -6.257 |
| NPC283316 | -6.032 |
| NPC249078 | -5.719 |
| NPC67337 | -5.676 |
| NPC109862 | -5.634 |
| NPC179897 | -5.554 |
| NPC319486 | -5.523 |
| NPC214153 | -5.411 |
| NPC235175 | -5.273 |
| Paeonol | -5.082 |
| NPC54339 | -4.949 |
